# Omega-3 Polyunsaturated Fatty Acids (n-3 PUFAs) in Cardiovascular Diseases (CVDs) and Depression: The Missing Link?

**DOI:** 10.1155/2009/725310

**Published:** 2009-09-27

**Authors:** Jane Pei-Chen Chang, Yi-Ting Chen, Kuan-Pin Su

**Affiliations:** ^1^Department of Psychiatry, China Medical University Hospital, Taichung 40447, Taiwan; ^2^Graduate Institute of Neural and Cognitive Sciences, China Medical University, Taichung 40447, Taiwan

## Abstract

*Background*. Based on epidemiological data, clinical trials, and meta-analytic reviews, omega-3 polyunsaturated fatty acids (n-3 PUFAs) seem to be a biological link between depression and cardiovascular diseases (CVDs). *Presentation*. Involvement of n-3 PUFAs in depression and CVDs may be associated with a chronic, low-grade, inflammation. We hypothesize that n-3 PUFAs link depression and CVDs via “PUFA-prostaglandin E2 (PGE2) cascade.” *Testing*. To further support our hypothesis, case-control studies are needed to test the role of COX2 and PLA2 functions in depression and in CVDs. In addition, the effects of n-3 PUFAs on cardiovascular markers in depression and on depressive symptoms in CVDs should be investigated in clinical trials. Finally, the effects of manipulating COX2 and PLA2 functions on depression-like behaviors and cardiovascular functions could be explored in animal studies. *Implications*. n-3 PUFAs might be a promising treatment for both cardiovascular diseases and depression via its anti-inflammatory, cardioprotective, and neuroprotective effects.

## 1. Background

### 1.1. The Missing Link from “Sadness” to “Heart-Breaking”

When people are upset or being hurt emotionally, we often describe them to have a “broken heart.” In Mandarin, we also refer the concept of “*sadness*” to “*sung-shin* (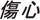),” which literally means “*heart-breaking*.” Although this metaphor by referring a dysfunctioning brain to a breaking heart is misleading biologically, accumulating evidence from empirical studies reveal that there seems to be a “mind-body interface” linking between cardiovascular diseases (CVDs) and depression.

### 1.2. The “Linking” between Depression to CVD

Depression and CVD are two highly comorbid diseases with 15 years of scientific evidence supporting this phenomenon [1]. Depression has an estimated 10% life prevalence rate in the general population, while an estimated of 17–27% prevalence rate of depression is noted in population with CVD [2]. In addition, depression observed following CVD is common and associated with increased risk of mortality. Depression is a potential prognostic factor to increase future cardiovascular event risk by 2–7.5 folds in patients with CVD [3]. One would expect that depression is the psychological response to a cardiovascular event; however, depression without any cardiovascular comorbidity has been found to increase the odds ratio (OR) for future CVD event (OR = 4.5) in the 13-year prospective study in Maryland Epidemiological Catchment Area [4]. Glassman et al. showed that heart rate variability (HRV) is an indicator reflecting fluctuations in autonomic activity and moderately strong and independent predictor of death, also, recovery after acute coronary syndrome was not observed in patients with major depressive disorder (MDD) [1]. Medically healthy individuals who suffer from depression are also at significantly increased risk of developing heart attacks and strokes later in life [5]. These findings imply that there might be a common pathway between depression and CVD.

The exact mechanisms interplaying between depression and CVD are still under investigation, however, clinical and interventional studies have shown that the bidirectional relation of the two are connected via adversely affected autonomic and hormonal homeostasis, which result in inflammation, metabolic abnormalities, hypercoagulability, and endothelial dysfunction [6]. Low-grade inflammation is one possible common mechanism responsible for the relationship between CVD and depression. Inflammatory process mediators such as arachidonic acid (AA) and its metabolites, prostaglandins (PGs) and leukotrienes (LTs), contribute to diverse circulatory and homeostatic functions [7]. PGs and LTs are highly biologically active, have proinflammatory action, vasoconstriction action, and are known to be involved in various pathological processes, such as atherosclerosis and CVD [8]. In patients with major depression, the inflammatory biomarkers including PGE2, IL-1, IL-6, and IL-12 have been found to be significantly increased as compared with healthy controls [9]. The role of inflammation in depression has also been demonstrated in animal models when endotoxin (lipopolysaccharide; LPS) or interleukin-1 (IL-1) is administered to induce sickness behavior that resembles depression [9]. In addition, depression is more frequently seen in those with medical disorders associated with immune dysfunction, for example, diabetes mellitus [10] and hepatitis C (HCV) patients treated with interferon alpha [9]. 

HPA axis hyperactivity has been reported as another possible mechanism to be associated with major depression and CVD [11]. Patients with major depression has been found to have elevated corticotrophin releasing factor (CRF) concentrations in cerebrospinal fluid (CSF) [12], blunting of adrenocorticotropic hormone (ACTH) response to CRF administration, nonsuppression of cortisol secretion following dexamethasone administration, and hypercortisolemia [13]. HPA axis hyperactivity in depression is also shown by dysregulation of multidrug resistance p-glycoprotein (MRD PGP), a membrane steroid transporter in the brain located on blood-brain-barrier [14]. Overactive MRD PGP in depressed patients reduces the access of glucocorticoids to brain and induces glucocorticoid resistance [15]. Administered corticosteroids have long been known to induce hypercholesterolemia, hypertriglyceridemia, and hypertension, which simulate the HPA hyperactivity condition in CVD patients [11]. The dysregulations of blood lipids and blood pressure predispose and exacerbate CVD. Studies have shown that elevated morning plasma cortisol concentrations have been significantly correlated with moderate-to-severe coronary atherosclerosis in young and middle-aged men [16].

## 2. Presentation of the Hypothesis

### 2.1. n-3 PUFAs in Depression and CVD

There are two main types of bioactive polyunsaturated fatty acids (PUFAs), the omega-6 (n-6) series (cis-linoleic acid [LA,18 : 2], *γ*-linolenic acid [GLA, 18 : 3, n-6], dihomo-GLA [20 : 3, n-6], arachidonic acid [AA, 20 : 4, n-6]), and the omega-3 (n-3) series (*α*-linolenic acid [ALA, 18 : 3], eicosapentaenoic acid [EPA, 20 : 5, n-3], docosahexaenoic acid [DHA]). However, n-3 and n-6 PUFAs are important constituents of all cell membranes and essential for survival of humans and other mammals. Because the n-3 PUFAs cannot be synthesized in the body and can only be obtained from our diet, they are also called essential fatty acids [9].

Based on the evidence from epidemiological data, case-controlled studies, and clinical trials, n-3 PUFAs have been found to be important in the development of depression and CVD. In epidemiological studies, it has been observed that societies with high consumption of n-3 PUFAs appear to have lower prevalence of CVD, as well as the prevalence of depression [17]. In case-controlled studies, lower levels of n-3 PUFAs have been found in both depression [18] and CVD patient groups [19]. Besides, the level of n-3 PUFAs is significantly negatively correlated with the severity of depressive symptoms [20] and they may act as both prognostic and diagnostic utility in CVD risk assessments [21]. n-3 PUFAs have antidepressant and antiarrhythmic effects, as revealed in basic and clinical studies of depressed patients [22] and CVD patients [23]. Meta-analysis of omega-3 on CVD with 228 864 individuals suggests that increase in fish intake, which is abundant with n-3 PUFAs, was associated with 20% significant (*P* < .005) reduction in the risk of fatal CVD and a significant (*P* < .005) 10% reduction in total CVD [24]. Many clinical studies showed that diet of n-3 PUFAs (especially EPA and/or DHA) could decrease the risk of CVD [25].

If n-3 PUFAs play an important role in depression and CVD, the enzymes for n-3 PUFA metabolisms might also have effects on these two diseases. Phospholipase A2 (PLA2) and cyclooxygenase 2 (COX2) are the two key enzymes of the PUFA metabolism and PGE2 synthesis [26]. PLA2 is a large family of enzymes, with Ca2+-independent phospholipase A2 (iPLA2) preferentially on DHA metabolism and cytolic PLA2 (cPLA2) preferentially on AA and EPA metabolism [27, 28]. CPLA2 cleaves PUFAs into free PUFAs and lysophospholipids, which can modulate signal transduction, transcriptional regulation, neuronal activity, apoptosis, and a number of other processes within the central nervous system [29], and the excess activity of the cPLA2 could lower PUFA response [30]. BanI polymorphism is one of the two gene polymorphisms of cPLA2 in chromosome 1q25, near the promoter region and first intron [31]. Genetic studies have revealed that the G allele of BanI polymorphism of cPLA2 increases the risk of developing depression in a Korean population [32] and the risk for depression among interferon alpha-treated HCV patient groups [33]. COX2 converts AA to PGE2, and PGE2 relates to development of depression and CVD via its actions in immunomodulation [34]. A functional G → C polymorphism located 765 basepairs upstream from the transcription start site (−765G → C) has been identified in the human COX2 gene with C allele leading to decreased promoter activity in vitro [35]. Studies have shown that C allele might protect against clinical events, for example, myocardial infarction (MI), stroke [36], and cerebrovascular ischemia [37]. C allele may also be associated with lower levels of inflammatory markers such as C-reactive protein and interleukin-6 in cardio-/cerebrovascular and hypercholesterolemic patients [38]. The C allele of COX2 gene polymorphism has been found to increase risk for CVD in the Finnish men population [39]. To our knowledge, the role of COX2 polymorphisms in depression and the role of PLA2 polymorphisms in CVD have not been confirmed yet.

### 2.2. Role of n-3 PUFAs in Depression and CVD

#### 2.2.1. Inflammation

**Figure 1:**
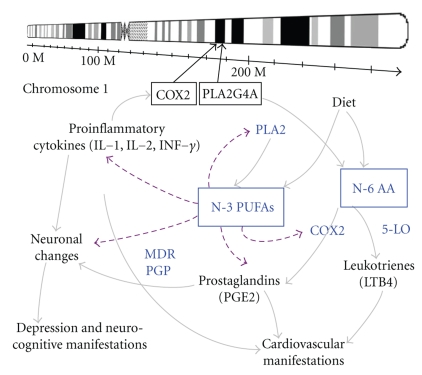
*Genetic and environmental factors related to n-3 fatty acids hypothesis of depression and CVD*. The levels of n-3 PUFAs (n-3 EPA and n-3 DHA) are influenced by genetic (e.g., PLA2 and COX2 genes on chromosome 1) and environmental (diet, inflammation, or cytokines) factors. n-3 DHA plays a major role in neuronal membrane stability and functions of signal transduction and neurotransmission; meanwhile, n-3 PUFAs are important in balancing the immune and inflammatory functions by antagonizing membrane n-6 AA and reducing PGE2 synthesis. PLA2 and COX2 are the two key enzymes for the PUFA metabolism and PGE2 synthesis. PLA2 is a large family of enzymes, with the iPLA2 (Ca2+-independent PLA2) preferentially functioning in n-3 DHA metabolism and the cPLA2 (cytosolic PLA2) preferentially in n-6 AA and n-3 EPA metabolism. COX2 is the key enzyme that converts n-6 AA to PGE2, while 5-LO converts n-6 AA to LTB4. PGE2 and LTB4 participate in immunoregulation, which might be associated with somatic symptoms of depression and physical manifestations of CVD. Proinflammatory cytokines, such as IL-2 and IFN-*γ*, activate PLA2 or COX2 and in turn increase levels of n-6 AA. MDR PGP has effects in depression by reducing the access of glucocorticoids to the brain. n-3 PUFAs, on the other hand, can inhibit MDR PGP. Enhancement is shown by a solid line, attenuation by a dashed line. COX2 = cyclooxygenase 2; PLA2 = phospholipase A2; 5-LO = 5-lipoxyge; MDR PGP = multidrug resistance p-glycoprotein; CVD = cardiovascular disease.

The PUFAs themselves appear to be active in the biological function, while some of their functions require their conversion to eicosanoids and other products. Figure 1 demonstrates how n-3 and n-6 PUFAs affect the pathogenesis of depression and CVD and how n-3 and n-6 PUFAs levels are influenced by genetic and environmental factors. The iPLA2 enzyme associates with n-3 DHA metabolism and cPLA2 enzyme participates in metabolism of n-6 AA and n-3 EPA [40]. N-6 AA converts to proinflammatory cytokines (PGE2 and LTB4) via COX2 and 5-lipooxygenase (5-LO), in turn, may contribute to the development of somatic symptoms in depression and the physical manifestation of CVD [40]. On the other hand, n-3 DHA might be connected to the etiology of mood and cognitive dysfunction in depression via its role in neuronal membrane stability, neuroplasticity, and neurotransmission [40]. Proinflammatory cytokines, such as IL-1, IL-2, and Interferon gamma (IFN-*γ*), have been extensively reported in their effects on activities of PLA2 or COX2 and levels of n-6 PUFA [41]. Consequently, the activation of PLA2 or COX2 can induce the release of AA from the membrane phospholipid [41], and n-3 PUFAs can reduce the activation of cPLA2 and the release of n-6 AA and PGE 2 induced by IL-1 [42]. In brief, the n-6 AA can form eicosanoid series (e.g., PGE2 and LTB4), which has proinflammatory, proaggregatory, and vasoconstrictive effect. n-3 PUFAs can antagonize n-6 PUFAs and produce oppositional biological effects.

#### 2.2.2. HPA Axis Hyperactivity

Deficiency in the n-3 PUFAs is associated with increased CSF corticotrophin releasing hormone (CRH), and contributes to HPA axis hyperactivity [43]. Animal studies show that the restoration of dietary DHA normalizes the exaggerated distress behavior of n-3 PUFAs deficient rats during administration of CRH [44]. Double-blind placebo-controlled intervention trials with human subjects have also demonstrated benefit of stress protection with n-3 PUFAs dietary supplements. n-3 PUFAs were shown to attenuate stress-induced increase in aggression and hostility among Japanese students in one study [45] and significantly reduced perceived stress among stressed university staff in the other study [46]. Neminen et al. in 2006 found that lower long chain omega-3 essential fatty acid status was associated with higher neuroactive steroids, such as 3*α*, 5*α*-tetrahydrodeoxycorticosterone (THDOC) which appear to counter-regulate HPA hyperactivity and concentrations in human subjects [47]. HPA axis hyperactivity is enhanced through MDR PGP overactivity, which reduces the access of glucocorticoids to the brain and is found to contribute to neuronal changes that might lead to depression [14]. n-3 PUFAs are able to antagonize the action of proinflammatory PGE2 effect, and in turn, normalize MDR PGP overactivity and HPA hyperactivity.

#### 2.2.3. Other Mechanisms

Stroke is largely associated with depression and CVD in recent studies. One perspective is that HPA axis hyperactivity contributes to risks for CVD by the states of hypercholesterolemia, hypertriglyceridemia, hypertension [11] and sympathoadrenal hyperactivity. As microvascular changes contribute to risks for CVD by the states of hypercholesterolemia, hypertriglyceridemia, hypertension, and sympathoadrenal hyperactivity [11], stroke or cerebrovascular lesions might also be involved in the pathogenesis of depression, in particular, the late-life vascular depression. On the other hand, patients with depression are associated with a prothrombotic state of hypercortisolemia and changes in platelet function related to HPA axis hyperactivity, which may consequently increase risks for CVD and stroke. n-3 PUFAs were shown to reduce mortality of stroke in recent interventional studies, such as GISSI [48] and DART study [49]. n-3 PUFAs' cardioprotection effects have also been supported by the findings showing an inverse relation between n-3 PUFAs intake and stroke [50]. Therefore, omega-3 PUFAs may also help to fill out the pieces among depression, stroke, and CVD. The role of n-3 PUFAs in other mechanisms, including antiangiogenesis and neurogensis of depression and CVD would need more studies to examine.

## 3. Testing the Hypothesis

Since there are many studies showing that n-3 PUFAs are promising interface connecting depression and CVD, more clinical and basic studies are warrant for completing the whole picture. Firstly, the role of COX2 polymorphisms in depression and the role of PLA2 polymorphisms in CVD need to be tested in case-control studies in clinical settings. Although Pae et al. have shown higher frequency of G allele of the PLA2 gene BanI polymorphism in 63 patients with MDD compared with 117 healthy controls in a Korean population [32]. As a direction for future studies, the result needs replication in a larger sample. In addition, the effect of COX2 polymorphisms on MDD has never been tested. In addition, the genetic effects of COX2 and PLA2 as well the therapeutic effects of n-3 PUFAs on specific symptoms, for example, depressive symptoms in CVD or somatic symptoms, such as HRV, chest tightness, dyspnea, somatic pain symptoms in depression, are also very important to support our hypothesis. Secondly, specific enzyme activities can influence the metabolism and synthesis of individual PUFAs (e.g., iPLA2 preferentially on DHA metabolism and cPLA2 preferentially on AA and EPA metabolism) [27, 28]. The role of iPLA2 and cPLA2 on depression and CVD should be clarified in future studies. Finally, by animal studies, one would be able to explore whether (1) COX2 enhancers would increase depression-like symptoms in mice and (2) PLA2 blockers would increase CVD risks in mice. If the results from basic study support the hypothesis of the role of COX2 in depression and the role of PLA2 in CVD, we will be able to take the step further into genetic studies and explore whether (1) mice of knockout cPLA2 genes would have increased the risk of CVD, since more studies have focused on cPLA2 gene polymorphism and its association with depression; and whether (2) mice of knockout COX2 genes would have increased depression-like behaviors in animal models.

## 4. Implications of the Hypothesis

By 2020, pointed out the World Health Organization, “depression will be second only to heart disease as a cause of disability and premature death in established market economies” [51]. n-3 PUFAs may link depression and cardiovascular diseases based on numerous data about its effects on immunomodulation and normalization of HPA axis functions. Hopefully, by accumulating more evidence from future larger-scale studies, n-3 PUFAs might be used as a remedy to cure one's depression and mend one's broken heart.

## References

[1] Glassman AH, Bigger JT, Gaffney M (2007). Heart rate variability in acute coronary syndrome patients with major depression, influence of sertraline and mood improvement. *Archives of General Psychiatry*.

[2] Evans DL, Charney DS, Lewis L (2005). Mood disorders in the medically ill: scientific review and recommendations. *Biological Psychiatry*.

[3] Jiang W, Glassman A, Krishnan R, O'Connor CM, Califf RM (2005). Depression and ischemic heart disease: what have we learned so far and what must we do in the future?. *American Heart Journal*.

[4] Eaton WW, Fogel J, Armenian HK, Eaton WW (2006). The consequences of psychopathology in the Baltimore epidemiologic catchment area follow-up. *Medical and Psychiatric Comorbidity over the Life Span*.

[5] Glassman AH, Bigger JT, Gaffney M (2006). Onset of major depression associated with acute coronary syndromes: relationship of onset, major depressive disorder history, and episode severity to sertraline benefit. *Archives of General Psychiatry*.

[6] Lippi G, Montagnana M, Favaloro E, Franchini M (2009). Mental depression and cardiovascular disease: a multifaceted, bidirectional association. *Seminars in Thrombosis and Hemostasis*.

[7] Gerritsen ME (1996). Physiological and pathophysiological roles of eicosanoids in the microcirculation. *Cardiovascular Research*.

[8] Das UN (2006). Essential fatty acids: biochemistry, physiology and pathology. *Biotechnology Journal*.

[9] Das UN (2007). Is depression a low-grade systemic inflammatory condition. *American Journal of Clinical Nutrition*.

[10] Musselman DL Medical illness and depression: a delicate interplay between biology and brain.

[11] Musselman DL, Evans DL, Nemeroff CB (1998). The relationship of depression to cardiovascular disease. *Archives of General Psychiatry*.

[12] Nemeroff CB, Widerlov E, Bissette G (1984). Elevated concentrations of CSF corticotropin-releasing factor-like immunoreactivity in depressed patients. *Science*.

[13] Raadsheer FC, van Heerikhuize JJ, Lucassen PJ (1994). Increased numbers of corticotropin-releasing hormone expressing neurons in the hypothalamic paraventricular nucleus of depressed patients. *Neuroendocrinology*.

[14] Murck H, Song C, Horrobin D, Uhr M (2004). Ethyl-eicosapentaenoate and dexamethasone resistance in therapy-refractory depression. *International Journal of Neuropsychopharmacology*.

[15] Pariante CM (2006). The glucocorticoid receptor: part of the solution or part of the problem?. *Journal of Psychopharmacology*.

[16] Troxler RG, Sprague EA, Albanese RA, Fuchs R, Thompson AJ (1977). The association of elevated plasma cortisol and early atherosclerosis as demonstrated by coronary angiography. *Atherosclerosis*.

[17] Hibbeln JR, Nieminen LR, Blasbalg TL, Riggs JA, Lands WE (2006). Healthy intakes of n-3 and n-6 fatty acids: estimations considering worldwide diversity. *American Journal of Clinical Nutrition*.

[18] Peet M, Murphy B, Shay J, Horrobin D (1998). Depletion of omega-3 fatty acid levels in red blood cell membranes of depressive patients. *Biological Psychiatry*.

[19] Mitsuhiro Y, Hideki O, Masunari M (2007). Effects of eicosapentaenoic acid on major coronary events in hypercholesterolameic patients(JELIS): a randomised open-label, blinded endpoint analysis. *The Lancet*.

[20] Edwards R, Peet M, Shay J, Horrobin D (1998). Omega-3 polyunsaturated fatty acid levels in the diet and in red blood cell membranes of depressed patients. *Journal of Affective Disorders*.

[21] Harris WS, Assaad B, Poston WC (2006). Tissue omega-6/omega-3 fatty acid ratio and risk for coronary artery disease. *American Journal of Cardiology*.

[22] Lin PY, Su KP (2007). A meta-analytic review of double-blind, placebo-controlled trials of antidepressant efficacy of omega-3 fatty acids. *Journal of Clinical Psychiatry*.

[23] Kang JX, Leaf A (1996). Antiarrhythmic effects of polyunsaturated fatty acids. Recent studies. *Circulation*.

[24] Whelton SP, He J, Whelton PK, Muntner P (2004). Meta-analysis of observational studies on fish intake and coronary heart disease. *American Journal of Cardiology*.

[25] Albert CM, Campos H, Stampfer MJ (2002). Blood levels of long-chain n-3 fatty acids and the risk of sudden death. *The New England Journal of Medicine*.

[26] Rao JS, Lee HJ, Rapoport SI, Bazinet RP (2008). Mode of action of mood stabilizers: is the arachidonic acid cascade a common target?. *Molecular Psychiatry*.

[27] Strokin M, Sergeeva M, Reiser G (2003). Docosahexaenoic acid and arachidonic acid release in rat brain astrocytes is mediated by two separate isoforms of phospholipase A2 and is differently regulated by cyclic AMP and Ca^2+^. *British Journal of Pharmacology*.

[28] Strokin M, Sergeeva M, Reiser G (2004). Role of Ca^2+^-independent phospholipase A2 and n-3 polyunsaturated fatty acid docosahexaenoic acid in prostanoid production in brain: perspectives for protection in neuroinflammation. *International Journal of Developmental Neuroscience*.

[29] Kam PC, See AU (2000). Cyclo-oxygenase isoenzymes: physiological and pharmacological role. *Anaesthesia*.

[30] Noponen M, Sanfilipo M, Samanich K (1993). Elevated PLA2 activity in schizophrenics and other psychiatric patients. *Biological Psychiatry*.

[31] Tay A, Simon JS, Squire J, Hamel K, Jacob HJ, Skorecki K (1995). Cytosolic phospholipase A2 gene in human and rat: chromosomal localization and polymorphic markers. *Genomics*.

[32] Pae CU, Yu HS, Kim JJ (2004). BanI polymorphism of the cytosolic phospholipase A2 gene and mood disorders in the Korean population. *Neuropsychobiology*.

[33] Su KP, Peng CY, Cheng JC, Pariante CM (2007). Polymorphisms in cytosolic phopholipase A2 and cyclooxygenase 2 genes and risk of interferon induced depression. *European Neuropsychopharmacology*.

[34] Su KP (2008). Mind-body interface: the role of n-3 fatty acids in psychoneruoimmunology, somatic presentation, and medical illness comorbidity of depression. *The Asia Pacific Journal of Clinical Nutrition*.

[35] Papafili A, Hill MR, Brull DJ (2002). Common promoter variant in cyclooxygenase-2 represses gene expression: eidence of role in acute-phase inflammatory response. *Arteriosclerosis, Thrombosis, and Vascular Biology*.

[36] Cipollone F, Toniato E, Martinotti S (2004). A polymorphism in the cyclooxygenase 2 gene as an inherited protective factor against myocardial infarction and stroke. *Journal of the American Medical Association*.

[37] Colaizzo D, Fofi L, Tiscia G (2006). The COX-2 G/C-765 polymorphism may modulate the occurrence of cerebrovascular ischemia. *Blood Coagulation and Fibrinolysis*.

[38] Orbe J, Beloqui O, Rodriguez JA, Belzunce MS, Roncal C, Paramo JA (2006). Protective effect of the G-765C COX-2 polymorphism on subclinical atherosclerosis and inflammatory markers in asymptomatic subjects with cardiovascular risk factors. *Clinica Chimica Acta*.

[39] Huuskonen KH, Tarja AK, Minna MT (2008). Research article: COX-2 gene promoter polymorphism and coronary artery disease in middle-aged men: the Helsinki sudden death study. *Mediators of Inflammation*.

[40] Su KP (2009). The biological mechanism of antidepressant effect of omega-3 fatty acids: how does fish oil acts as a “mind-body interface?”. *NeuroSignals*.

[41] Wu T, Levine SJ, Lawrence MG, Logun C, Angus CW, Shelhamer JH (1994). Interferon-*γ* induces the synthesis and activation of cytosolic phospholipase A2. *The Journal of Clinical Investigation*.

[42] Song C, Li X, Kang Z, Kadotomi Y (2007). Omega-3 fatty acid ethyl-eicosapentaenoate attenuates IL-1beta-induced changes in dopamine and metabolites in the shell of the nucleus accumbens: involved with PLA2 activity and corticosterone secretion. *Neuropsychopharmacology*.

[43] Hibbeln JR, Bissette G, Umhau JC, George DT (2004). Omega-3 status and cerebrospinal fluid corticotrophin releasing hormone in perpetrators of domestic violence. *Biological Psychiatry*.

[44] Takeuchi T, Iwanaga M, Harada E (2003). Possible regulatory mechanism of DHA-induced anti-stress reaction in rats. *Brain Research*.

[45] Hamazaki T, Sawazaki S, Itomura M (1996). The effect of docosahexaenoic acid on aggression in young adults: a placebo-controlled double-blind study. *The Journal of Clinical Investigation*.

[46] Bradbury J, Myers SP, Oliver C (2004). An adaptogenic role for omega-3 fatty acids in stress; a randomized placebo controlled double blind intervention study (pilot). *Nutrition Journal*.

[47] Nieminen LRG, Makino KK, Mehta N, Virkkunen M, Kim HY, Hibbeln JR (2006). Relationship between omega-3 fatty acids and plasma neuroactive steroids in alcoholism, depression and controls. *Prostaglandins Leukotrienes and Essential Fatty Acids*.

[48] Marchioli R, Barzi F, Bomba E (2002). Early protection against sudden death by n-3 polyunsaturated fatty acids after myocardial infarction: time-course analysis of the results of the gruppo italiano per lo studio della sopravvivenza nell'Infarto miocardico (GISSI)-Prevenzione. *Circulation*.

[49] Burr ML, Fehily AM, Gilbert JF (1989). Effects of changes in fat, fish, and fibre intakes on death and myocardial reinfarction: diet and reinfarction trial (DART). *The Lancet*.

[50] He K, Rimm EB, Merchant A (2002). Fish consumption and risk of stroke in men. *Journal of the American Medical Association*.

[51] Murray CJ, Lopez AD (1996). *The Global Burden of Disease: A Comprehensive Assessment of Mortality and Disability from Diseases, Injuries, and Risk Factors in 1990 and Projected to 2020*.

